# Chiropractic spinal manipulative therapy for cervicogenic headache: a study protocol of a single-blinded placebo-controlled randomized clinical trial

**DOI:** 10.1186/s40064-015-1567-5

**Published:** 2015-12-16

**Authors:** Aleksander Chaibi, Jūratė Šaltytė Benth, Peter J. Tuchin, Michael Bjørn Russell

**Affiliations:** Head and Neck Research Group, Research Centre, Akershus University Hospital, 1478 Lørenskog, Norway; Institute of Clinical Medicine, Akershus University Hospital, University of Oslo, 1474 Nordbyhagen, Norway; HØKH, Research Centre, Akershus University Hospital, Lørenskog, Norway; Department of Chiropractic, Macquarie University, Sydney, NSW 2109 Australia

**Keywords:** Cervicogenic headache, Headache, Randomized controlled trial, Chiropractic, Spinal manipulation, Manual therapy, Protocol

## Abstract

Cervicogenic headache (CEH) is a secondary headache which affects 1.0–4.6 % of the population. Although the costs are unknown, the health consequences are substantial for the individual; especially considering that they often suffers chronicity. Pharmacological management has no or only minor effect on CEH. Thus, we aim to assess the efficacy of chiropractic spinal manipulative therapy (CSMT) for CEH in a single-blinded placebo-controlled randomized clinical trial (RCT). According to the power calculations, we aim to recruit 120 participants to the RCT. Participants will be randomized into one of three groups; CSMT, placebo (sham manipulation) and control (usual non-manual management). The RCT consists of three stages: 1 month run-in, 3 months intervention and follow-up analyses at the end of intervention and 3, 6 and 12 months. Primary end-point is headache frequency, while headache duration, headache intensity, headache index (frequency × duration × intensity) and medicine consumption are secondary end-points. Primary analysis will assess a change in headache frequency from baseline to the end of intervention and to follow-up, where the groups CSMT and placebo and CSMT and control will be compared. Due to two group-comparisons, the results with p values below 0.025 will be considered statistically significant. For all secondary end-points and analyses, the significance level of 0.05 will be used. The results will be presented with the corresponding p values and 95 % confidence intervals. To our knowledge, this is the first prospective manual therapy three-armed single-blinded placebo-controlled RCT to be conducted for CEH. Current RCTs suggest efficacy in headache frequency, duration and intensity. However a firm conclusion requires clinical single-blinded placebo-controlled RCTs with few methodological shortcomings. The present study design adheres to the recommendations for pharmacological RCTs as far as possible and follows the recommended clinical trial guidelines by the International Headache Society.

*Trial registration* ClinicalTrials.gov identifier: NCT01687881, 2 December 2012

## Background

The prevalence of cervicogenic headache (CEH) is low and varies from 1.0 to 4.6 % in the general population, depending on the applied diagnostic criteria, i.e. 1.0 % if 6 and 4.6 % if 5 diagnostic criteria of the Cervicogenic Headache International Study Group are fulfilled, and 2.5 % if the criteria of the International Headache Society (IHS) are applied (Table 1) (Nilsson 1995a; Pareira Monteriro 1995; Sjaastad et al. 1998; Sjaastad and Bakketeig 2008; Headache Classification Subcommittee of the International Headache Society 2013). Headache disorders have substantial health and socio-economic costs (Vos et al. 2012). However, no studies have exclusively investigated the costs for CEH.

**Table 1:** Diagnostic criteria for cervicogenic headache by the Cervicogenic Headache International Study Group

Major criteria^a^
1. Symptoms and signs of neck involvement
a. Precipitation of head pain, similar to the usually occurring one:
i. By neck movement and/or sustained awkward head positioning, and/or:
ii. By external pressure over the upper cervical or occipital region on the symptomatic side
b. Restriction of range of motion (ROM) in the neck
c. Ipsilateral neck, shoulder, or arm pain of a rather vague nonradicular nature or, occasionally, arm pain of a radicular nature.
2. Confirmatory evidence by diagnostic anesthetic blockade
3. Unilaterality of the head pain, without side shift
Head pain characteristics
4. Moderate-severe, non-throbbing, and non-lancinating pain, usually starting in the neck. Episodes of varying duration, or: fluctuating, continuous pain
Other characteristicsof some importance
5. Only marginal effect or lack of effect of indomethacin. Only marginal effect or lack of effect of ergotamine and sumatriptan. Female sex. Not infrequent occurrence of head or indirect neck trauma by history, usually of more than only medium severity.
Other features of lesser importance
6. Nausea. Phonophobia and photophobia. Dizziness. Ipsilateral “blurred vision”. Difficulties swallowing. Ipsilateral oedema, mostly in the periocular area

Diagnostico criteria for cervicogenic headache by the International Classification of Headache Disorders-II
A. Pain, referred from a source in the neck and perceived in one or more regions of the head and/or face, fulfilling criteria C and D
B. Clinical, laboratory and/or imaging evidence of a disorder or lesion within the cervical spine or soft tissues of the neck known to be, or generally accepted as, a valid cause of headache
C. Evidence that the pain can be attributed to the neck disorder or lesion based on at least one of the following:
i Demonstration of clinical signs that implicate a source of pain in the neck
ii Abolition of headache following diagnostic blockade of a cervical structure or its nerve supply using placebo- or other adequate controls
D. Pain resolves within 3 months after successful treatment of the causative disorder or lesion

^a^It is obligatory that one or more of phenomena 1a–c are present

CEH is a symptomatic headache characterized by primarily chronic and usually unilateral headache as well as symptoms and signs of neck involvement (Sjaastad et al. 1998; Sjaastad and Fredriksen 2000; Bogduk and Govind 2009). CEH is often worsened by neck movement, sustained awkward head position, external pressure over the upper cervical or occipital region on the symptomatic side (Pareira Monteriro 1995; Sjaastad and Fredriksen 2000). Abolition of the headache following diagnostic anaesthetic blocks of cervical structures or local factors in the neck gives evidence that the pain is attributed to a neck disorder or lesion (Pareira Monteriro 1995; Sjaastad and Bakketeig 2008; Bogduk and Govind 2009).

Pharmacological management is the first treatment option for CEH despite the limited effect. The risk of medication overuse due to frequent headache attacks represents therefore a major health hazard and both direct and indirect cost concerns. The prevalence of medication overuse headache (MOH) is 1–2 % in the general population (Grande et al. 2008; Aaseth et al. 2008; Jensen and Stovner 2008), i.e. about half the population suffering chronic headache (15 headache days or more per month) have MOH (Lundqvist et al. 2012).

Diversified technique and Gonstead method are the two most commonly used chiropractic manipulative treatment modalities in the profession, used by 91 and 59 % of chiropractors, respectively (Cooperstein 2003; Cooperstein and Gleberson 2004), along with other manual and non-manual interventions, i.e., soft tissue techniques, spinal and peripheral mobilization, rehabilitation, postural corrections and exercises as well as general nutrition and dietetic advises.

A few spinal manipulative therapy (SMT) randomized controlled trials (RCTs) using the Diversified technique have been conducted for CEH, suggesting an effect on headache frequency, headache duration, headache intensity and medicine consumption (Nilsson 1995b; Nilsson et al. 1997; Jull et al. 2002; Haas et al. 2004, 2010; Borusiak et al. 2010). However, common for previous RCTs are the methodological shortcomings, such as; inaccurate headache diagnosis, i.e., questionnaire-based diagnoses used are imprecise (Rasmussen et al. 1991), inadequate or no randomization procedure involved, lack of placebo group, and primary and secondary end-points not pre-specified (Vernon 1995; Chaibi and Russell 2012; Fernandez-de-las-Penas et al. 2006). In addition, previous RCTs mostly included participants with infrequent CEH attacks and did not adhere to the guidelines from the IHS (Tfelt-Hansen et al. 2000; Silberstein et al. 2008). At present, no RCTs have applied the Gonstead method. Thus, considering the methodological shortcomings in previous RCTs, a clinical placebo-controlled RCT with improved methodological quality remains to be conducted for CEH.

Although CEH is the headache we best understand, the SMT mechanism of action is unknown. It is argued that CEH might originate from a complexity of nociceptive afferent responses involving the upper cervical spine (C1, C2 and C3), leading to a hypersensitivity state of the trigeminal pathway conveying sensory information for the face and much of the head (Kerr 1972; Bogduk 2004). Research has thus, suggested that SMT may stimulate neural inhibitory systems at different spinal cord levels and might activate various central descending inhibitory pathways (McLain and Pickar 1998; Vernon 2000; Vicenzino et al. 2001; Boal and Gillette 2004; De Camargo et al. 2011). However, although the proposed physiological mechanisms are not fully understood, there are likely additional unexplored mechanisms which could explain the effect that SMT has on mechanical pain sensitisation.

The objective is to investigate the efficacy of CSMT vs. placebo (sham manipulation) and controls (continue usual pharmacological management without receiving manual intervention) for CEH in a RCT.

## Methods and design

This is a single-blinded placebo-controlled RCT with three parallel groups (CSMT, placebo and control). Our primary hypothesis is that CSMT gives at least 25 % reduction in average number of headache days per month (30 days) as compared to no change in the placebo and the control group from baseline to the end of intervention, and we expect the same reduction to maintain at 3, 6 and 12 months follow-up. If the CSMT treatment is effective, it will be offered to participants whom received placebo or control after study completion, i.e., after 12 months follow-up. The study will adhere to the recommended clinical trial guidelines from the IHS (Tfelt-Hansen et al. 2000; Silberstein et al. 2008), and the methodological CONSORT guidelines (Moher et al. 2010).

### Patient population

Participants will be recruited in the period September to October 2012 through Akershus University Hospital and Innlandet Hospital Trust, Norway. Participants will receive posted information about the project followed by a short telephone interview.

Eligible participants are between 18 and 70 years of age and have at least one headache attack per month. Participants are diagnosed according to at least three major criteria not including occipital nerve blockage from the diagnostic criteria of the Cervicogenic Headache International Study Group (Sjaastad et al. 1998; Knackstedt et al. 2010). All participants are diagnosed by a neurologist.

Exclusion criteria are contraindication to SMT, spinal radiculopathy, pregnancy, depression and CSMT within the previous 12 months. Participants whom during the RCT receive any manual interventions by physiotherapists, chiropractors, osteopaths or other health professionals to treat musculoskeletal pain and disability, including massage therapy, joint mobilization and manipulation (French et al. 2011), changed their prophylactic headache medicine or pregnancy will be withdrawn from the study at that time and be regarded as drop-outs.

In response to initial contact, participants fulfilling the inclusion criteria will be invited to further assessment by the chiropractic investigator. The assessment includes an interview and a physical examination with special emphasis on the whole spinal column. Oral and written information about the project will be provided in advance and written consent will be obtained from all accepted participants during the interview by the clinical investigator. In accordance with good clinical practice, all patients will be informed about the harms and benefits as well as possible adverse reactions of the intervention primarily including local tenderness and tiredness on the treatment day. No serious adverse events have been reported for the chiropractic Gonstead method (Cassidy et al. 2008; Tuchin 2012). Participants randomized into active or placebo interventions, will undergo a full spine radiographic examination and be scheduled for 12 intervention sessions. The control group will not be exposed to this assessment.

### Clinical randomized controlled trial

**Fig. 1:**
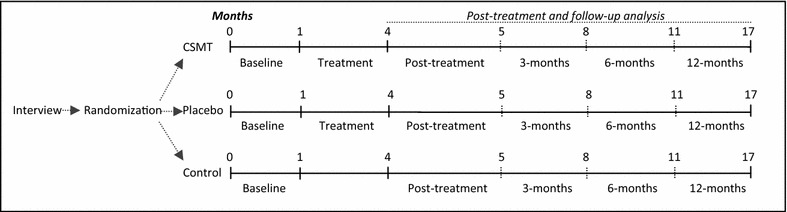
Flow-chart of study

The clinical RCT consists of 1 month run-in and 3 months intervention. Time profile from baseline to the end of follow-up will be assessed (Fig. 1).

### Run-in

The participants will fill in a validated diagnostic paper headache diary one month prior to intervention which will be used as baseline data for all participants (Russell et al. 1992; Lundqvist et al. 2009). The validated diary includes questions directly related to the primary and secondary end-points. X-rays will be taken in standing position in the anterior–posterior and lateral planes of the entire spine. The X-rays will be assessed by the clinical investigator.

### Randomization

Prepared sealed lots with the three interventions, i.e., active treatment, placebo and the control group, will be divided into four sub-groups by age and gender, i.e., woman and men, 18–39 and 40–70 years, respectively. Participants will be equally allocated to one of the three arms by only allowing participants to draw one lot. The blocked randomization will be administrated by an external trained party without the involvement from the clinical investigator.

### Intervention

Active treatment consists of CSMT using the Gonstead method (Cooperstein 2003). A specific contact, high-velocity, low-amplitude, short-lever, with no recoil post spinal adjustment directed to spinal biomechanical dysfunction diagnosed by standard chiropractic tests.

The placebo intervention consists of sham manipulation, i.e., a broad non-specific contact, low-velocity, low-amplitude sham push manoeuvre in a non-intentional and non-therapeutic directional line. All the non-therapeutic contacts will be performed outside the spinal column with adequate joint slack and without pre-tension so no joint cavitations occur (Chaibi et al. 2015). In some sessions, the participant lay either prone on a Zenith 2010 HYLO bench with the investigators standing at the participant’s right side with his left palm placed on the participant’s right lateral scapular edge with the other hand reinforcing. In other sessions, the investigator will stand at the participant’s left side and place his right palm over the participant’s left scapular edge with the other hand reinforcing, delivering a non-intentional lateral push manoeuvre. Alternatively, the participant lay in the same side posture position as the active treatment group with the bottom leg straight and top leg flexed with top leg’s ankle resting on the bottom leg’s knee fold, in preparation for a side posture push move, which will be delivered as a non-intentional push in the gluteal region. The sham manipulation alternatives will be equally interchanged among the placebo participant’s according to protocol during the 12 weeks treatment period to strengthen the study validity. Both the active and the placebo group will receive the same structural and motion assessment prior to and after each intervention. No additional co-interventions or advises will be given to participants during the trial period. The treatment period will include 12 consultations, i.e., twice per week the first three weeks followed by once a week the next two and once every second week until 12 weeks are reached. Fifteen minutes will be allocated per consultation for each participant. All interventions will be administered by an experienced chiropractor (AC) at Akershus University Hospital and Innlandet Hospital Trust, Norway.

The control group will continue usual care, i.e., pharmacological management without receiving manual intervention by the clinical investigator. The same exclusion criteria apply for the control group during the whole study period.

### Blinding

Participants whom receive active or placebo intervention will fill in a de-blinding questionnaire after each of the 12 treatment sessions administrated by an external trained independent party with no involvement from the clinical investigator, i.e., providing a dichotomous “yes” or “no” answer as to the question whether active treatment was received. This response was followed by a second question regarding how certain they were that active treatment was received on a 0–10 numeric rating scale (NRS), where 0 represents absolutely uncertain and 10 represents absolute certainty. The control group and the clinical investigator can for obvious reasons not be blinded (Bang et al. 2004; Johnson 2005; Chaibi et al. 2015).

### Follow-up

Follow-up analysis will be conducted on the end-points measured after the end of intervention and 3, 6 and 12 months follow-up. During this period all participants will continue to fill in a diagnostic headache diary and return it on a monthly basis. In the case of unreturned diary or missing values in the diary, the participants will be contacted immediately upon detection to minimize recall bias. Participants will be contacted by phone to secure compliance.

### Primary and secondary end-points

The primary and secondary end-points are listed in Table 2. The end-points adhere to the recommended IHS clinical trial guidelines (Tfelt-Hansen et al. 2000; Silberstein et al. 2008). We define number of headache days to be primary end-point and expect at least 25 % reduction in average number of days from baseline to the end of intervention, with the same level of reduction maintaining at follow-up. Based on previous reviews on CEH, a 25 % reduction is considered to be a conservative estimate (Chaibi and Russell 2012). A 25 % reduction from baseline to the end of intervention and follow-up is also expected in secondary end-points for headache duration, headache intensity, and headache index, where the index is calculated as mean days with headache (30 days) × mean headache duration (hours per day) × mean intensity (0–10 NRS). A 50 % reduction in medication consumption from baseline to the end of intervention and to follow-up is expected.

**Table 2:** Primary and secondary end-points

Primary end-points
1. Number of headache days in active treatment vs. placebo group
2. Number of headache days in active treatment vs. control group
Secondary end-points
3. Headache duration in hours in active treatment vs. placebo group
4. Headache duration in hours in active treatment vs. control group
5. Self-reported VAS in active treatment vs. placebo group
6. Self-reported VAS in active treatment vs. control group
7. Headache index (frequency × duration × intensity) in active treatment vs. placebo group
8. Headache index in active treatment vs. control group
9. Headache medication dosage in active treatment vs. placebo group
10. Headache medication dosage in active treatment vs. control group

The data analysis is based on the run-in period vs. end of intervention. Point 11–40 will be duplicate of point 1–10 above at respectively 3, 6 and 12 months follow-up

### Data processing

**Fig. 2:**
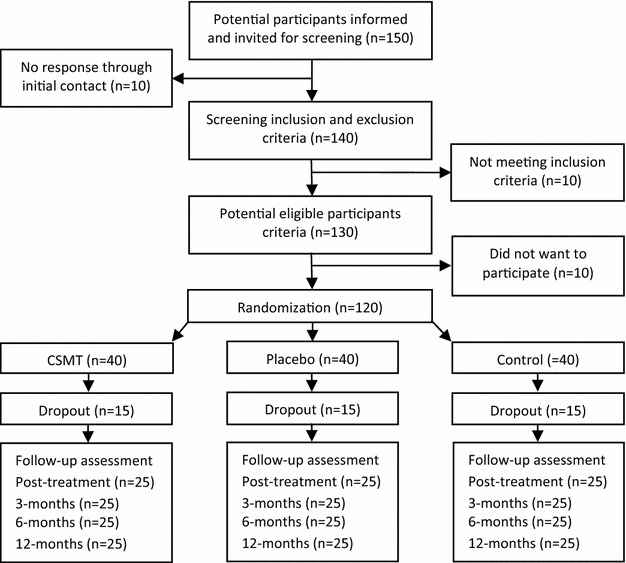
Expected participants flow diagram

A flowchart of the participants is shown in Fig. 2. Baseline demographic and clinical characteristics will be tabulated as means and standard deviations (SD) for continuous variables and proportions and percentages for categorical variables. Each of three groups will be described separately. Primary and secondary end-points will be presented by suitable descriptive statistics in each group and for each time point. Normality of end-points will be assessed graphically and transformation will be considered if necessary.

Change in primary and secondary end-points from baseline to the end of intervention and to follow-up will be compared between active and placebo and active and control group. Null-hypothesis states that there is no significant difference between the groups in average change, while the alternative hypothesis states that a difference of at least 25 % exists.

Due to follow-up period, repeated recordings of primary and secondary end-points will be available, and analyses of trend in primary and secondary end-points will be of main interest. Intra-individual correlations (cluster effect) are likely to be present in data with repeated measurements. Cluster effect will thus be assessed by an intra-class correlation coefficient (ICC) quantifying the proportion of total variation attributable to the intra-individual variations. The trend in end-points will be assessed by a linear regression model for longitudinal data (linear mixed model) to correctly account for possible cluster effect. Linear mixed model handles unbalanced data, enabling all available information from randomized patients to be included, also from drop-outs. Regression models with fixed effects for time component and group allocation as well as the interaction between the two will be estimated. The interaction will quantify possible differences between groups regarding time trend in the end-points and serve as an omnibus test. Random effects for patients will be included to adjust the estimates for intra-individual correlations. Random slopes will be considered. The linear mixed models will be estimated by SAS PROC MIXED procedure. The two pairwise comparisons will be performed by deriving individual time point contrasts within each group with the corresponding p values and 95 % confidence intervals (CI).

Both per-protocol and intention-to-treat analyses will be conducted if relevant. All analyses will be performed by a statistician, blinded for group allocation and participants. All adverse effects will also be registered and presented. Participants who experience any sort of adverse effects during the trial period will be entitled to call the prime investigator on the project cell phone. The data will be analyzed with SPSS v22 and SAS v9.3. Because of two group-comparisons in a primary end-point, p values below 0.025 will be considered statistically significant. For all secondary end-points and analyses, a significance level of 0.05 will be used. Missing values might appear in incomplete interview questionnaires, incomplete headache diaries, missed intervention sessions and/or due to drop-outs. The pattern of missingness will be assessed and missing values handled adequately.

### Power calculation

Sample size calculations are based on the results in a recently published group comparison study on topiramate (Silberstein et al. 2004). We hypothesize that the mean difference in reduction of number of days with headache per month between active and the placebo group is 2.5 days. The same difference is assumed between active and the control group. Standard deviation for reduction in each group is assumed to be equal 2.5. Under the assumption of on average 10 headache days per month at baseline in each group and no change in the placebo or control group during the study, 2.5 days reduction corresponds to a reduction by 25 %. As primary analysis includes two group-comparisons, we set a significance level at 0.025. A sample size of 20 patients is required in each group to detect a statistical significant mean difference in reduction of 25 % with 80 % power. To allow for drop-outs, the investigators plan to recruit at least 120 participants.

### Ethics

The study has been approved by the Norwegian Regional Committee for Medical Research Ethics (REK) (2010/1639/REK) and the Norwegian Social Science Data Services (11–77). The declaration of Helsinki is otherwise followed. All data will be anonymised while participants must give oral and written informed consent, including consent to publish all obtained data. Insurance is provided through “The Norwegian System of Compensation to Patients” (NPE) which is an independent national body, set up to process compensation claims from patients who have suffered an injury as a result of treatment under the Norwegian health service. A stopping rule is defined for withdrawing participants from this study in accordance with recommendations in the CONSORT extension for Better Reporting of Harms (Ioannidis et al. 2004). If a participant reports to their chiropractor or research staff a severe adverse event, he or she will be withdrawn from the study and referred to their General Practitioner or hospital emergency department depending on the nature of the event. Data will be stored in a locked cabinet at the Research Centre, Akershus University Hospital, Norway, for 5 years.

## Discussion

### Methodological considerations

Current SMT RCTs on CEH suggest treatment efficacy regarding headache frequency, headache duration and headache intensity. However, a firm conclusion requires clinical single-blinded placebo-controlled RCTs with few methodological shortcomings (Chaibi and Russell 2012). Such studies should adhere to the recommended IHS clinical trial guidelines with headache frequency as primary end-point and headache duration, headache intensity, headache index and medication consumption as secondary end-point (Tfelt-Hansen et al. 2000; Silberstein et al. 2008). Headache index, combination of frequency, duration and intensity, gives an indication of the total level of suffering. Headache index has despite the lack of consensus been recommended as an accepted standard secondary end-point, thus, we included this as a secondary outcome (Bendtsen et al. 1996; Silberstein et al. 2008; Hagen et al. 2009). The primary and secondary end-points will be collected prospectively in a validated diagnostic headache diary for all participants in order to minimize recalling bias (Russell et al. 1992; Lundqvist et al. 2009).

To our knowledge, this is the first prospective manual therapy three-armed single-blinded placebo-controlled RCT to be conducted for CEH. The study design adheres to the recommendations for pharmacological RCTs as far as possible. RCTs that include a placebo and control group are advantageous to pragmatic RCTs that compare two active treatment arms. RCTs also provide the best approach for producing safety as well as efficacy data.

An unsuccessful blinding is a possible risk to the RCT. Blinding is often difficult as there is no single validated standardized chiropractic sham intervention which can be used as a control group to this date. It is however, necessary to include a placebo group in order to produce a true net effect of the active intervention. Consensus about an appropriate placebo for a clinical trial of SMT among experts representing both clinicians and academics has, however, not be reached (Hancock et al. 2006). No previous studies have to our knowledge, validated a successful blinding of a CSMT clinical trial with multiple treatment sessions. We intend to minimize this risk by following the proposed protocol for the placebo group.

The placebo response is furthermore high in pharmacological and assumed similarly high for non-pharmacological clinical studies, however, it might be higher in manual therapy RCTs were attention and physical contact is involved (Meissner et al. 2013). Similarly, a natural concern with regards to attention bias will be involved for the control group as they are not being seen by anyone or not seen as much as the other two groups.

There are always risks for drop-outs due to various reasons. As the trial duration is 16 months with a 12 months follow-up period, the risk for loss to follow-up is thus enhanced. Co-occurrence of other manual intervention during the trial period is another possible risk, as those whom receive manipulation or other manual physical treatments elsewhere during the trial period will be withdrawn from the study and regarded as drop-outs at the time of violation. Participants are allowed to continue their usual medication throughout the trial.

The external validity of the RCT might be a weakness as there is only one investigator. However, we found that advantageous to multiple investigators, in order to provide similar information to participants in all three groups and manual intervention in the CSMT and the placebo group. Thus, we intend to eliminate inter-observer variability which might be present if there are two or more investigators. Although the Gonstead method is the second most used technique among chiropractors, we do not see an issue of concern when it comes to generalizability and external validity.

Recruiting participants to the study might however be a challenge due to the low prevalence in the general population and our strict exclusion criteria. A weak representativeness of the study population might thus influence the results and generalizability.

The internal validity is, however, strong by having one investigator. It reduces the risk of potential selection, information and experimental biases. Furthermore, all participants are diagnosed by an experienced neurologist and not by questionnaires. A direct contact through interview has higher sensitivity and specificity as compared to questionnaire (Rasmussen et al. 1991). Individual motivational factors which can influence participant’s perception as well as personal preferences when treating are both reduced by having one investigator. In addition, the internal validity is further strengthened by a concealed validated randomization procedure.

Conducting X-rays prior to the active and placebo interventions was found applicable in order to visualize posture, joint and disc integrity (Taylor 1993; International Chiropractic Assocoation Practicing Chiropractors’ Committee on Radiology Protocols (PCCRP) for biomechanical assessment of spinal subluxation in chiropractic clinical practice. 2009). As the total X-ray radiation dose varies from 0.2–0.8 mSv, the radiation exposure was considered low (Cracknell and Bull 2006; Borretzen et al. 2007). X-ray assessments were also found necessary in order to determine if full spine X-rays are useful in future studies or not.

As we are unaware of the mechanisms of possible efficacy, and both spinal cord and central descending inhibitory pathways has been postulated, we see no reasons to exclude a full spine treatment approach for the intervention group. It has furthermore been postulated that pain in different spinal regions should not be regarded as separate disorders but rather a single entity (Leboeuf et al. 2012). Similarly, including a full spine approach limits the differentiations between the CSMT and the placebo group. Thus, it might strengthen the likelihood of successful blinding in the placebo group being achieved. In addition, all the placebo contacts will be performed outside the spinal column, thus, minimizing a possible spinal cord afferent input.

### Innovative and scientific value

This RCT will highlight and validate the Gonstead CSMT for CEH, which has not previously been studied. If CSMT proves to be efficient, it will provide a non-pharmacological treatment option. This is especially important as pharmacological management for CEH has no or only minor effect. Thus, if CSMT works, it can really have an impact on CEH treatment. The study also bridges cooperation between chiropractors and physicians, which is important in order to make the healthcare more efficient. Finally, our method might be applied in future chiropractic and other manual therapy RCTs on headache.

## Abbreviations

CEH: cervicogenic headache
CI: confidence interval
CSMT: chiropractic spinal manipulative therapy
HVLA: high velocity low amplitude
ICC: intra-class correlation coefficient
IHS: international headache society
MOH: medication overuse headache
mSv: milliSievert
NPE: The Norwegian system of compensation to patients
NRS: numeric rating scale
RCT: randomized controlled trial
REK: Norwegian regional committee for medical research ethics
SD: standard deviation
SMT: spinal manipulative therapy
